# Lower cell number, lateral defect location and milder grade are associated with improved autologous chondrocyte implantation outcome

**DOI:** 10.1002/ksa.12433

**Published:** 2024-08-27

**Authors:** Lauren Tierney, Jan H. Kuiper, Sally Roberts, Martyn Snow, Mike Williams, Mateus B. Harrington, Paul Harrison, Pete Gallacher, Paul Jermin, Karina T. Wright

**Affiliations:** ^1^ School of Pharmacy and Bioengineering, Centre for Regenerative Medicine Research Keele University Staffordshire UK; ^2^ Oswestry Keele Orthopaedic Research Group Robert Jones and Agnes Hunt Orthopaedic Hospital Oswestry Shropshire UK; ^3^ Division of Psychological Medicine & Clinical Neurosciences Cardiff University Cardiff UK

**Keywords:** autologous chondrocyte implantation, knee cartilage injury, longitudinal modelling, patient‐reported outcome measures

## Abstract

**Purpose:**

To investigate patient demographic, injury and surgery/treatment‐associated factors that can influence the patient‐reported outcome (Lysholm score), following autologous chondrocyte implantation (ACI) in a large, ‘real‐world’, nonuniform, prospective data examined retrospectively.

**Methods:**

Knee patients treated at the Robert Jones and Agnes Hunt Orthopaedic Hospital, UK, using ACI between 1996 and 2020 were eligible. All longitudinal postoperative Lysholm scores collected between 1 and 23 years after ACI treatment and before any second major procedure (e.g., arthroplasty) were included. Multilevel longitudinal models were built investigating the association of short‐term (1 year) or long‐term trends in Lysholm score with baseline demographic, clinical and cell‐culture variables, namely age, gender, smoker status, body mass index, baseline Lysholm score, time from surgery, defect grade, diameter and location, number of defects, previous microfracture, patch/scaffold type, associated procedure(s), number of cells implanted and their passage number.

**Results:**

Following filtering, 306 of the 427 knee ACI procedures reviewed were suitable for inclusion. Factors shown to result in higher postoperative Lysholm scores in the short term were lower patient age, higher baseline Lysholm scores, fewer implanted cells and a lateral femoral defect location. The factor which was associated with higher long‐term postoperative Lysholm scores was a milder defect grade. Additionally, the failure rate in this cohort was explored and it was found that 73/306 (24%) of patients experienced joint failure according to our definition. Furthermore, the outcome was not influenced by coincidental procedures in this cohort of patients.

**Conclusions:**

This study has identified a number of baseline factors associated with patient‐reported outcomes following ACI and shows that treatment of associated pathology at the time of surgery potentially restores patient outcomes to a similar level as those with no associated pathologies.

**Level of Evidence:**

Level IV.

**Abbreviations**
ACI: autologous chondrocyte implantation
AIC: Akaike Information Criterion
BMI: body mass index
ICRS: International Cartilage Regeneration and Joint Preservation Society
KOOS: Knee injury and Osteoarthritis Outcome Score
LFC: lateral femoral condyle
MACI: matrix‐assisted ACI
MFC: Medial Femoral Condyle
MSC: mesenchymal stromal cell
OA: osteoarthritis
PROM: patient‐reported outcome measure

## INTRODUCTION

Autologous chondrocyte implantation (ACI) provides relief for symptomatic cartilage defects and although the formation of hyaline cartilage is rare, ACI does result in the formation of functional repair tissue at the defect site [1, 2, 3, 4, 24, 28, 30, 32]. While most patients have positive clinical results, outcomes vary between individuals, meaning treatment failures and osteoarthritis (OA) are still a problem, despite many years of experience and modifications to the technique. Several studies have aimed to help predict which patients will demonstrate positive outcomes based on demographic, injury or surgery‐associated risk factors. A retrospective audit of patients who had received matrix‐assisted ACI (MACI) found that nonsmokers consistently demonstrated higher post‐ACI function (Cincinnati score) at 3 years, compared to smokers and ex‐smokers [17]. These findings were confirmed in patients following arthroscopic MACI [17]. Similarly, Jaiswal et al. [16] found significantly improved Cincinnati scores in patients with normal body weight, with 82% having good/excellent scores, compared to just 5.5% of patients in the obese group [16]. Furthermore, patients without concomitant pathologies have been reported to have significantly higher functional (Lysholm) scores at 6 and 12 months post‐MACI than those with them [12]. Additional factors reported in the literature are potentially influenced by selection bias based on surgeon selection criteria.

A systematic review of nine studies with 771 ACI patients and a mean follow‐up time of 11.4 years demonstrated that procedures performed on patients who were older or with defects >4.5 cm^2^ resulted in an increased risk of reoperation and failure [29]. This review defined failure as repeat ACI, conversion to osteochondral autologous transfer surgery or osteochondral allograft or unicompartmental or total knee arthroplasty. A 10‐year follow‐up study of 210 patients also found larger defects to be a risk factor for ACI failure, in addition to prior marrow stimulation [25]. A previous study exploring factors potentially predictive of knee arthroplasty following ACI (using some of the same patient data included in the current study) found that higher age, being female, a larger number of defects, a patellar defect, a history of previous operations in the joint being treated and a lower baseline Lysholm score increased the likelihood of knee replacement and used these to formulate a risk score distinguishing five risk categories [5]. Using revision surgery as an outcome, a more recent study also identified the female sex and in addition a body mass index (BMI) ≥ 35 as predictive factors [11].

The aim of the current study was to investigate the association of patient characteristics, surgical procedures, defect characteristics and growth kinetics of the implanted cells with both short‐term 12‐month and longitudinally collected post‐ACI Lysholm scores. The approach was to utilise a ‘real‐world’ data set to investigate (i) whether current proposed short‐ to mid‐term predictors of successful clinical outcome following ACI influence the long‐term outcome and (ii) whether new potential predictors could be identified from a large cohort of patients. The overarching goal was to identify a set of predictive factors that could add to the current ACI recommendations, for example, the UK National Institute for Health and Care Excellence guidance (TA477) [17]. To the best of our knowledge, this is the largest and longest follow‐up study of patients with very little refinement criteria.

## MATERIALS AND METHODS

The study was ethically approved by the Black Country Research Ethics Committee (REACT 09/H1203/90). Knee patients treated using ACI between 1996 and 2020 at the Robert Jones and Agnes Hunt (RJAH) Orthopaedic Hospital were eligible for inclusion in the study and provided informed verbal and/or written consent. The inclusion criteria were any patient who had undergone standard chondrocyte ACI using a patch and did not present with any of the exclusion criteria (listed below). Information was extracted from the prospective Oscell database of cell therapy patients. Several hospital‐based resources were used to resolve missing baseline data, including paper records of surgery and cell manufacture facility procedures, clinical trial documents and electronic patient records. Patients were excluded if they had no available postoperative Lysholm score, or if they fell into a treatment group whereby they had:

(1) Received autologous cell treatment by direct injection of cells into the joint or if mesenchymal stromal cells were used in the procedure.
(2) Undergone any type of bone graft or bone scaffold accompanying the ACI procedure, given the negative effect of subchondral bone abnormalities on ACI [1].
(3) Severely damaged knees due to major trauma such as a road traffic accident, where cartilage damage was not the main pathology.

The baseline information collected comprised age at ACI, sex, BMI, smoking status, prior microfracture, defect grade, diameter and location, number of defects, patch type (periosteum or collagen membrane), number of cells implanted, passage number and concurrent procedure(s). The primary outcome was a self‐administered version of the Lysholm Knee Scale [37], which was collected in the clinic at baseline and by mail annually after the procedure [37]. The total score was determined using weighting based on a Rasch analysis and converted to a score out of 100 for consistency with the original Lysholm [36]. The test–retest reliability of the Lysholm, assessed using the intraclass correlation coefficient (ICC), is 0.91 [20].

### Data refinement

The study aimed to analyse how the Lysholm score changed over time after ACI up to the point of failure. Failure was defined as the requirement for a major second procedure to address the previously treated joint surface, such as a repeat cartilage repair, osteotomy or total or partial joint replacement. Any scores completed after any of these procedures were excluded. If a patient had multiple defects, only characteristics for the largest treated defect (e.g., size, grade and location) were used in the analysis. Categories with less than five patients were combined to aid model performance, and their new combined label was used. The reference categories for each of the three categorical variables were Smoking status—‘yes’; Defect grade—‘International Cartilage Regeneration and Joint Preservation Society (ICRS) grade 3’; Defect location—‘Medial Femoral Condyle (MFC)’. Two approaches for representing the data on concurrent surgery were compared: [1] A binary variable indicating whether patients had or did not have a parallel procedure or [2] A categorical variable indicating the type of procedure that occurred alongside ACI, with ‘None’ being the reference category.

### Statistical analysis

All analyses were performed using R vs. 4.1.1 (2021‐08‐10) using the following packages: lme4 (1.1.27.1) to build the models, lmerTest (3.1.3) for backwards elimination, sjPlot (2.8.10) and jtools (2.1.4) for model outputs, lattice (0.20.44) and ggeffects (1.1.1) for plots. Unless reported otherwise, a *p* value below 0.05 was assumed to denote significance and mean values were reported with their 95% confidence intervals.

#### Handling missing data

Baseline characteristics that were missing were imputed. For categorical parameters such as smoking, the missing value was imputed as ‘unknown’. For continuous data such as BMI or baseline Lysholm, the mean value was imputed for missing values. There was no need to impute missing postoperative Lysholm values because the analysis method used (mixed‐effects modelling) naturally handles this type of missingness [8]. The level of imputation for each parameter is reported in the results.

#### Building linear mixed models: Random intercept and random slope models

Since the data were unbalanced and nonindependent, with multiple scores per patient at unequally spaced time points, linear mixed‐effects modelling was used [8] with Lysholm score as dependent, time as independent and patient as a clustering variable. The random slope and intercept model provided a better fit for the data accounting for the fact that each patient had both varying year 1 postoperative scores and long‐term trajectories [23, 39].

#### Selecting and generating the best‐fit linear mixed model

All baseline variables and their interaction with time were added as fixed independent variables to create a full model. Backward elimination of the fixed variables was used, aiming to achieve a minimal value for the Akaike Information Criterion (AIC) at each elimination step and implemented by using likelihood ratio tests at a fixed *p* value of 0.157 [14]. This method was used for both data representations on concurrent surgery (approaches 1 and 2, described earlier) and the representation giving the lowest AIC was taken forward. The assumption of normally distributed residual errors (difference between predicted and actual Lysholm score) in the final best‐fit model was checked using quantile–quantile plots. Symmetric deviations from a straight line at the top and tail end of the plot (‘heavy tails’) were considered acceptable [10]. The only effect of such deviations is to inflate standard errors and make tests for fixed effects more conservative, but this would not affect any of the main conclusions of the study [31].

#### Sample size estimation

A general rule for linear multivariable models is to have a sample size of approximately 15 times the number of potential independent variables [13]. However, a linear mixed‐effects model has a larger effective sample size because each patient has multiple measurements (Lysholm scores). The effective sample size depends on the number of measurements per patient and the within‐patient correlation between the measurements, the ICC [13]. For longitudinal patient‐reported outcome measure (PROM) measurements, the ICC is typically around 0.5 and assuming 5–10 measurements per patient the effective sample size is almost double the number of patients. Hence, assuming around 40 independent variables (20 intercepts and 20 slopes), one would need around 600/2 or 300 patients.

## RESULTS

A total of 411 patients (427 knees) were treated with ACI at the RJAH Orthopaedic Hospital between 1996 and 2020. Of these, 53 patients (knees) were excluded because no postoperative score was available, 61 because they had received a bone graft or scaffolding, four because they received direct injection or stem cells and three because they had a major trauma, leaving 290 patients (306 knees). Their mean baseline age was 37 years (SD 10) and 66% were male. The mean BMI was 28 (SD 5) and just over half did not smoke (52%). The mean defect diameter was 21 mm (SD 8), almost half of them on the MFC (46%). Sixty‐seven percent of patients had single defects, mostly ICRS grade 3 or 4. The mean baseline Lysholm score was 50 (SD 18; full details in Tables 1 and 2).

**Table 1:** Categorical baseline demographic and clinical data.

**Variable**	**Subgroups**	**Number per subgroup**	**Percentage**
Gender	Female	103	34
	Male	203	66
Smoker	Ex	11	4
	No	159	52
	Yes	30	10
	Unknown	106	35
Prior microfracture	No	174	57
	Yes	62	20
	Unknown	70	23
Concurrent procedure	No	162	53
	Yes	109	36
	Unknown	35	11
Defect location	LFC	56	18
	MFC	141	46
	LTP	12	4
	MTP	2	1
	Patella	48	16
	Trochlea	47	15
Defect grade	1	1	0
	2	13	4
	3	66	22
	4	99	32
	Unknown	127	41
Number of defects	Single	206	67
	Multiple	100	33
Passage number of implanted cells	1	44	14
	2	200	65
	3	53	17
	4	3	1
	Unknown	6	2
Patch type	Chondro‐Gide®	179	59
	Periosteum	121	40
	Other		
	Two patch types	2	1
	Unknown	4	1

*Note*: Where a subgroup has two subdivisions (e.g., defect location LTP and MTP), subgroups were combined for the model because at least one subgroup had fewer than five cases. The left subgroup denotes the combined one used in the model and the left denotes the actual subgroups in the data.

Abbreviations: LFC, lateral femoral condyle; LTP, lateral tibial plateau; MFC, Medial Femoral Condyle; MTP, Medial tibial plateau.

**Table 2:** Continuous baseline demographic and clinical data.

Age at the time of surgery	
Number	306
Number missing	0
Imputed %	0
Range	15–72.9 years
Mean	36.9 years
SD	9.9 years
BMI	
Number	266
Number missing	40
Imputed %	15.0
Range	18.4–45.9
Mean	28.0
SD	5.2
Defect diameter	
Number	296
Number missing	10
Imputed %	3.4
Range	4.5–58.1 (mm)
Mean	20.7 (mm)
SD	7.5 (mm)
Cells implanted	
Number	299
Number missing	7
Imputed %	2.3
Range	1–16 million
Mean	5.57 million
SD	2.03 million
Preoperative Lysholm score	
Number	280
Number missing	26
Imputed %	9.3
Range	12.5–91.7
Mean	49.9
SD	17.9

*Note*: number is the number of patients for whom data were available; number missing is the number of missing entries (imputed with mean value in the model).

Abbreviations: BMI, body mass index; SD, standard deviation.

**Figure 1:**
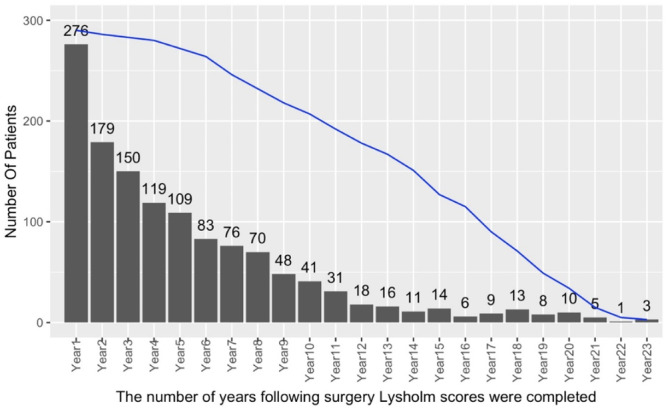
The number of patients who completed and returned Lysholm questionnaires per follow‐up year. The vertical axis shows the number of patients who completed and returned the Lysholm score per follow‐up year. The blue line represents the number of responses per year if all eligible patients (eligibility excludes those who were deceased) had completed and returned their Lysholm questionnaires, while the bars show the actual number of questionnaires returned and used for this analysis.

**Figure 2:**
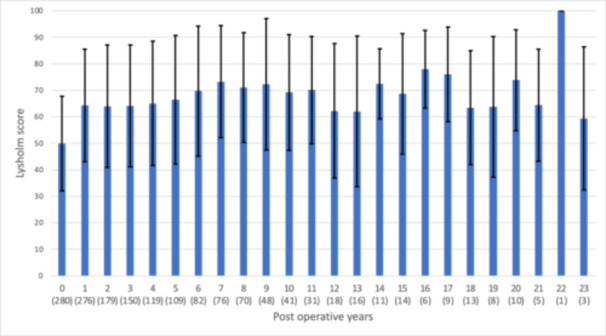
The mean postoperative Lysholm score per follow‐up year. The mean Lysholm score per year is shown with the standard deviation, where 0 on the *x* axis is the mean baseline score prior to surgery, with 1–23 being subsequent annual postoperative scores and the number of patient responses per follow‐up year is shown in brackets.

One year postsurgery, 276 patients (95%) returned a Lysholm questionnaire but the response rate gradually declined in subsequent years (Figure 1). The mean number of returned annual follow‐up scores was four but varied widely between patients (Supporting Information S1: Table 1). Sixty‐two patients returned only one post‐operative Lysholm score whereas 23 patients returned at least 10 annual postoperative scores, although not always in consecutive years. The mean postoperative Lysholm score was consistently greater than 58, over the explored 1–23 years following the patients' surgery (Figure 2). The data show narrow confidence intervals around the mean scores obtained in the first 5 years, whereas beyond 12 years postsurgery, there was a noticeable decline in follow‐up scores, with less than 20 responses per follow‐up year being received (Figure 1). The number of patients considered to experience joint failure was 73/306 (24%), with a mean time till joint failure of 6 years (SD 5).

### Random slope and intercept model outputs

Comparing the models with random intercept/fixed slope and random intercept and slope showed that introducing a random slope improved the model significantly (likelihood‐ratio test, *p* < 0.001, Supporting Information S1: Table 2).

### Generation of the best‐fit model

**Figure 3:**
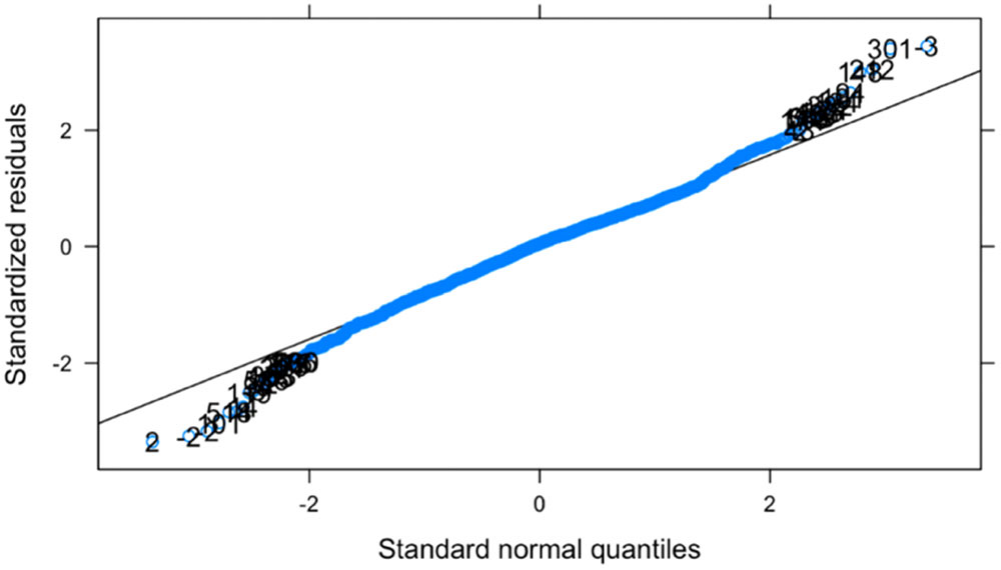
Q‐Q plot showing the normality of the residuals of the full model.

A full model was generated including all variables of interest and their interaction with time, to identify variables associated with short‐term change (1‐year) or longer‐term changes (up to 23 years) in Lysholm scores (Supporting Information S1: Table 3). Comparing the two approaches for representing concurrent procedures, the AIC was marginally lower when using binary coding (yes/no) and this method was, therefore, used. After backwards elimination, the final model comprised seven variables associated with 1‐year scores and four associated with the longer‐term trend (Table 3). The distribution of the residual errors was found to be approximately normal (Figure 3). Case identifiers 2 and 3 appeared to be strong outliers and were checked but their data were found to be correct.

**Table 3:** An output of each coefficient in the best‐fit model. The significant coefficients are highlighted in bold.

	Lysholm		
Variable	Estimates	CI	*p*
**(Intercept)**	**127.00** **a**	**46.57** **–** **207.43**	**0.002**
**Short** **‐** **term** **outcome (1 year)**			
**Age at the time of surgery**	**−0.21**	**−0.42 to −0.00**	**0.047**
Smoker	‐	‐	0.060
Smoker [ex]	−2.62	−14.97 to 9.73	0.677
Smoker [no]	6.89	−0.05 to 13.84	0.052
Smoker [unknown]	2.83	−4.42 to 10.07	0.444
Defect grade	‐	‐	0.651
Defect grade [1 and 2]	−5.32	−16.73 to 6.09	0.360
Defect grade [4]	0.94	−5.20 to 7.08	0.764
Defect grade [unknown]	1.61	−4.36 to 7.58	0.597
Defect location	‐	‐	0.073
**Defect location [LFC]**	**6.27**	**0.31** **–** **12.23**	**0.039**
Defect location [LTP and MTP]	−3.46	−14.62 to 7.71	0.544
Defect location [patella]	−3.72	−10.64 to 3.20	0.292
Defect location [trochlea]	3.89	−3.02 to 10.80	0.270
**Preoperative Score 100**	**0.47**	**0.34** **–** **0.61**	**<0.001**
**Cells to surgery log**	**−5.29**	**−10.56 to −0.02**	**0.049**
Passage number	‐	‐	0.109
Passage number [2]	−4.40	−11.05 to 2.26	0.195
Passage number [3 and 4]	1.16	−7.29 to 9.61	0.788
Passage number [unknown]	7.62	−8.05 to 23.28	0.340
***Long** **‐** **term** **trend***			
Time	−0.95	−2.23 to 0.33	0.147
**Time × Defect grade**	‐	‐	**0.013**
Time × Defect grade [1 and 2]	1.15	−0.47 to 2.77	0.165
**Time × Defect grade [4]**	**−0.96**	**−1.79 to −0.14**	**0.023**
**Time × Defect grade [unknown]**	**−0.83**	**−1.63 to −0.04**	**0.040**
Time × Defect location	‐	‐	0.068
Time × Defect location [LFC]	−0.36	−1.07 to 0.36	0.326
Time × Defect location [LTP and MTP]	0.32	−1.32 to 1.97	0.701
Time × Defect location [patella]	0.93	−0.13 to 1.99	0.085
Time × Defect location [trochlea]	−0.94	−1.97 to 0.09	0.074
Time × Preoperative Score 100	0.02	−0.00 to 0.03	0.062
Time × Passage number	‐	‐	0.146
Time × Passage number [2]	0.71	−0.03 to 1.46	0.061
Time × Passage number [3 and 4]	0.71	−0.24 to 1.66	0.144
Time × Passage number [unknown]	−0.43	−1.93 to 1.07	0.573
**Random effects**			
*σ* ^2^	173.37		
*τ*_00_	253.91		
*τ*_11_	1.54		
*ρ*_01_	−0.33		
ICC	0.60		
*N*	306		
Observations	1295		
Marginal *R* ^2^/conditional *R* ^2^	0.236/0.693		

Abbreviations: CI, confidence interval; ICC, intraclass correlation coefficient.

^a^ The intercept value of 127 points represents the score at Time = 0 that a patient would have if independent continuous variables have a value of zero (e.g., Age = 0, log(cell number) = 0, implying 1 cell) and categorical variables have their reference value (e.g., Smoker = Yes, Defect grade = 3, etc.). Given the age of the youngest patient [15] and the smallest number of cells implanted (1 million), the intercept value represents an extrapolated and, therefore, hypothetical number.

**Figure 4:**
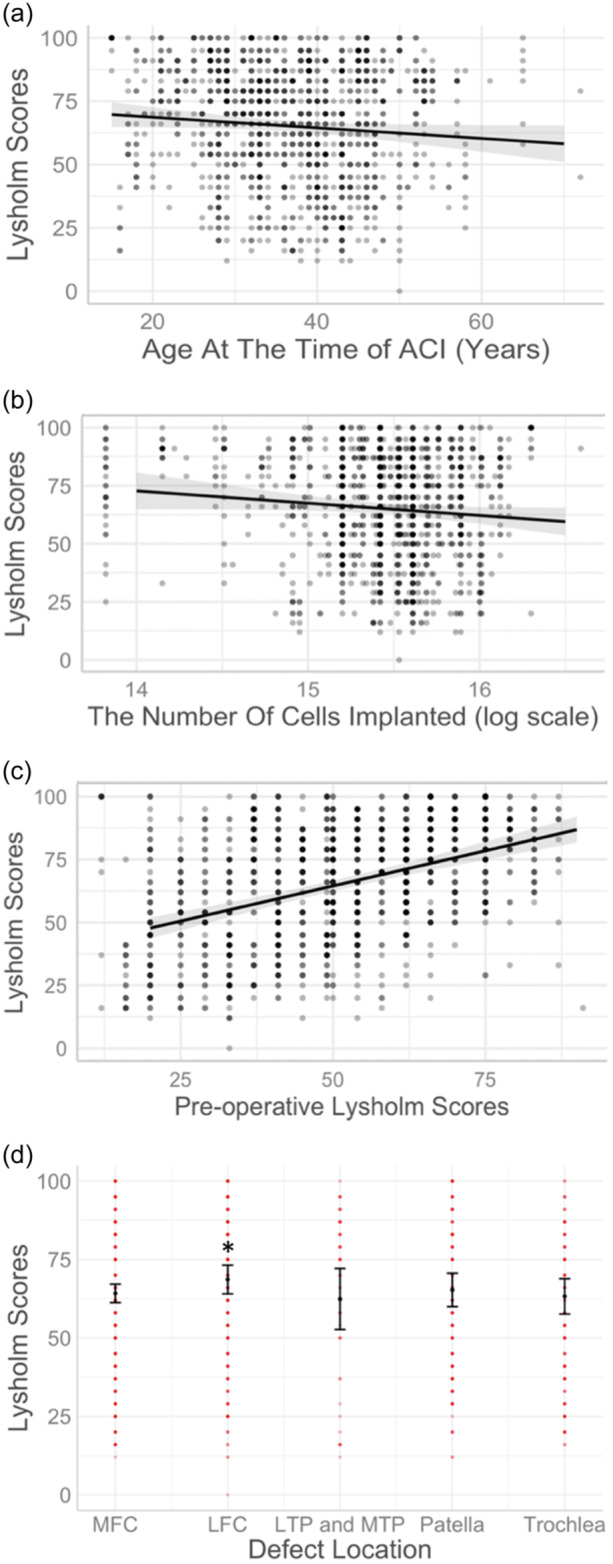
Variables that significantly influence 1‐year Lysholm scores. In all graphs, the vertical axis represents Lysholm scores 1‐year postsurgery. The horizontal axis displays the potential predictor variables: (a) Age at the time of autologous chondrocyte implantation (ACI); (b) number of cells implanted; (c) preoperative Lysholm scores; (d) defect location, patients with lateral femoral condyle (LFC) defects had significantly better Lysholm scores at 1‐year postsurgery compared to Medial Femoral Condyle (MFC) defects. *Significant difference between categories (MFC and LFC, *p* < 0.05). LTP, lateral tibial plateau; MTP, medial tibial plateau.

**Figure 5:**
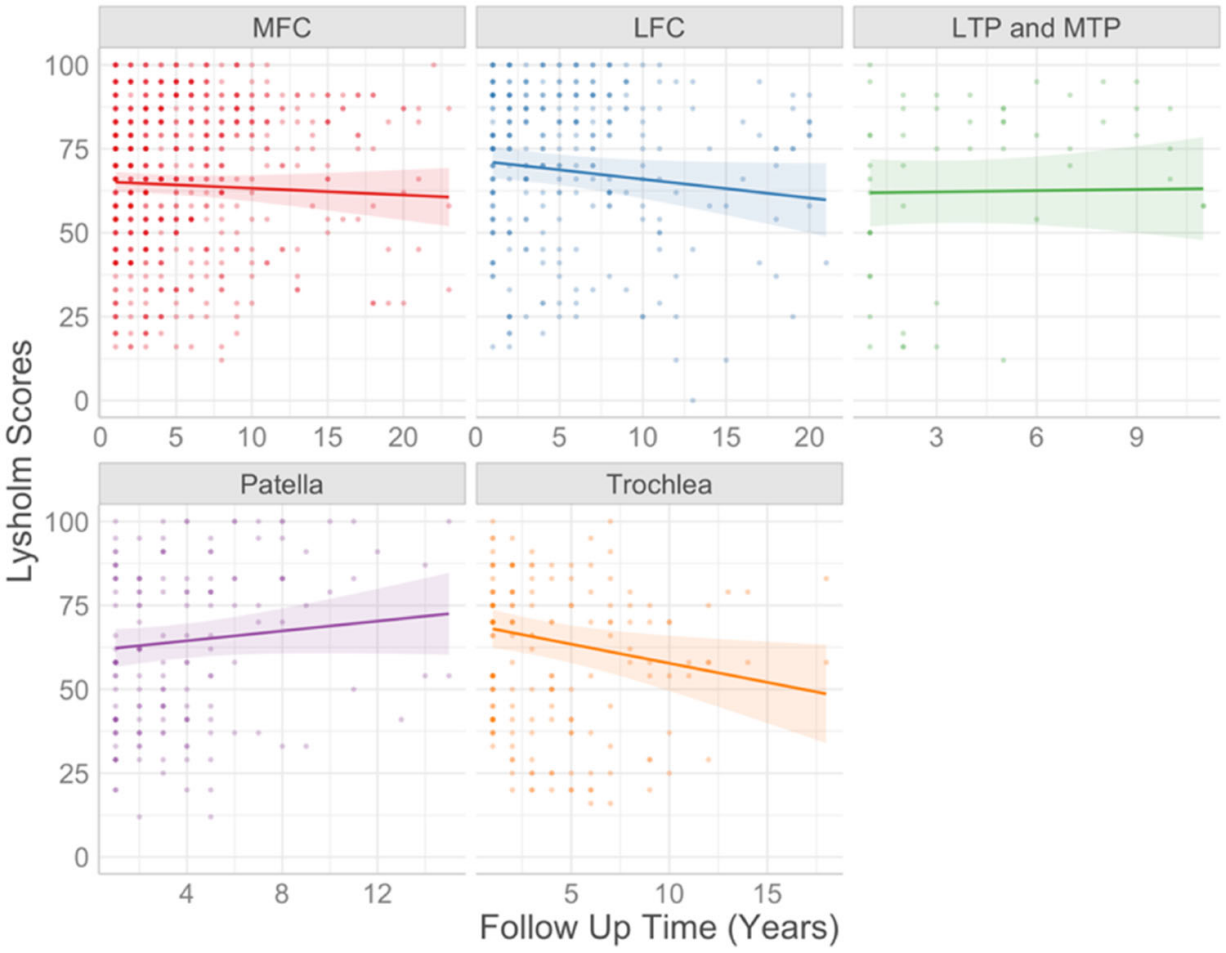
The effect of defect location on longitudinal Lysholm scores. This figure displays the long‐term trends in Lysholm scores for each of the defect locations. Medial Femoral Condyle (MFC) defects were set as the baseline and scores from other defect locations were compared to this. LFC, lateral femoral condyle; LTP, lateral tibial plateau; MTP, medial tibial plateau.

According to this final model, every year older at the time of surgery was associated with a mean decrease of 0.2 Lysholm points 1‐year postsurgery (*p* = 0.047; Figure 4a). Every unit increase in log cell number (equivalent to a 2.7‐fold increase) resulted in a 5.3‐point lower mean 1‐year score (*p* = 0.049; Figure 4b). Every point increase in preoperative Lysholm score was associated with a mean increase of 0.5 points at 1 year (*p* = <0.001; Figure 4c). Finally, patients with a lateral femoral condyle (LFC) defect had mean Lysholm scores that were 6.3 points higher 1‐year after surgery compared to patients with MFC defects (*p* = 0.039; Figure 4d). However, no evidence was found that having an LFC defect was associated with a different long‐term change in Lysholm over time. In contrast, patients with trochlear defects showed the greatest decline in Lysholm scores over time, but when compared against the longitudinal outcome of MFC defect patients, the difference was not significant (Figure 5). However, when using patients with patellar defects as the comparator group, the decline was significantly faster. Worse long‐term trajectories of trochlear defects were also found when defect location was used as a single independent variable and compared to both MFC defects (*p* = 0.023) (Supporting Information S1: Table 4) and patellar defects (*p* = 0.002) (Supporting Information S1: Table 5).

**Figure 6:**
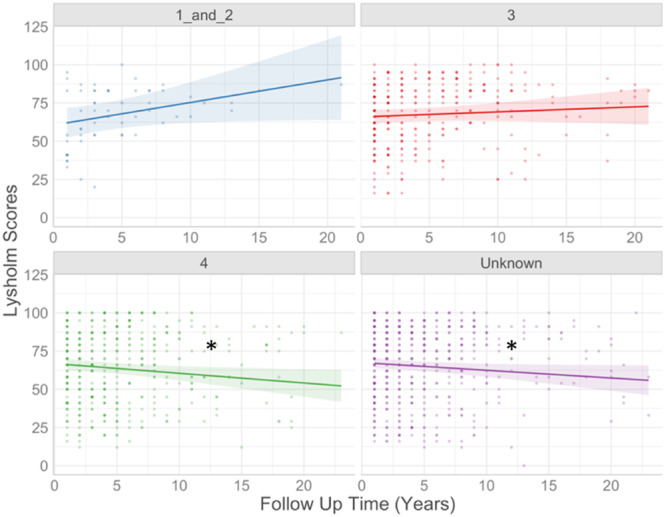
Influence of defect grade on trends in longitudinal Lysholm scores. The output of the model illustrated the variable that had a significant (*p* < 0.05) impact on patient‐reported Lysholm scores longitudinally. Defect grade was shown to significantly influence longitudinal Lysholm scores. Grade 3 was set as the baseline and all other grades were compared to this. *Significant difference between categories: Grades 3 and 4 (*p* < 0.05) and grade 3 and unknown defect grade.

Finally, defect grade was associated with longer‐term trends in Lysholm scores, with patients having a grade 4 defect losing on average 1.0 points per year more than patients with grade 3 defects (*p* = 0.023; Figure 6). Preoperative Lysholm scores did not significantly affect the longitudinal Lysholm scores.

## DISCUSSION

The main findings from this study are that a lower number of cells implanted, lower age at the time of ACI, higher preoperative Lysholm scores and a defect on the LFC were associated with better outcomes one year after surgery, while having a grade 4 defect was associated with a worse longer‐term trend in Lysholm scores compared to grade 3. The approach used provided a comprehensive analysis of clinically relevant data from a large number of ACI‐treated individuals who had a wide range of baseline demographic and clinical characteristics. Unusually cell characteristics (the number used and growth kinetics) were also examined.

Lysholm scores at 1‐year postsurgery were found to be 0.2 points lower for every added year in age. Poorer postoperative outcomes in older patients have been reported previously [21]. An analysis of data from seven individual studies with a minimum of 9 years of follow‐up found that there was a significant strong positive correlation between patient age and reoperation rate (*r* = 0.81, *p* = 0.001) [29]. Older patients are known to take longer to recover after surgery, which could explain why younger patients have higher scores at 1 year postoperatively whereas age does not significantly impact long‐term postoperative scores.

Patients with higher baseline Lysholm scores were found to have higher Lysholm scores at 1 year following surgery, similar to other studies [15], with each point a higher baseline score resulting in a mean 0.5 point higher 1‐year score. This is not surprising given that baseline and follow‐up PROMs in general show correlation coefficients around *r* = 0.5 [38]. Perhaps more surprising was that higher preoperative scores were only weakly associated with a more positive longer‐term longitudinal trend, equivalent to at most 0.3 points per extra preoperative Lysholm point over a decade (based on the upper 95% confidence limit). Conversely, patients with a lower baseline score have a more negative trend and may, therefore, fail earlier, akin to findings reported by Dugard et al. on 170 ACI‐treated patients in our centre, with up to 19 years of follow‐up, which found that a low preoperative Lysholm score was predictive of arthroplasty [5]. In this previous study, which included many of the same patients as the current study, a different panel of predictors was identified, but this is likely due to different outcome measures: longitudinal Lysholm scores in the current and survival in earlier study [5].

Interestingly, implanting a higher number of cells during the surgery was associated with lower Lysholm scores at 1 year, such that for every unit increase in log cell number, the Lysholm scores decreased by 5.3 points. A 2012 review of cell seeding densities in ACI found no studies addressing the effect of cell seeding density on clinical outcomes and concluded that there was no evidence that higher seeding densities led to better outcomes [9]. A later study on chondrocyte spheroids grouping 75 recipients into low, medium and high spheroid density found that all three groups had similar functional and imaging outcomes at 12 months [27]. However, treatment of OA in other cell dosing studies has shown the largest pain reduction in patients receiving the lowest doses [30]. An explanation for this result and our own findings could be that implanting a higher number of cells leads to greater competition for nutrients, increasing cell death and cancelling any potential gain in functional repair or tissue regeneration. Alternatively, implantating large numbers of cells might lead to nonuniform clumps of cells, which might have a negative impact on the repair. Whatever the reason(s), this study has shown an important association between smaller cell numbers implanted (likely equating to a ‘dose’ in pharmaceutical terms) and improved patient‐reported functional outcomes. This observation has novel and important implications for both regulatory authorities and cell therapy developers and manufacturers.

Having a defect on the LFC instead of the MFC was associated with a 6.3‐point better 1‐year Lysholm score. While this is not a clinically important difference, it is a significant difference and highlights the model's sensitivity in detecting small differences. The MFC was used as a baseline comparator as it tends to be the most frequent injury location in patients receiving ACI [19]. To our knowledge, no existing research indicates that outcomes of ACI differ between patients who have defects on the medial or LFC, making this a novel finding worthy of further investigation. The incidence of OA is known to be higher in the MFC [22]. A worse outcome with defects on the MFC might, therefore, indicate that ACI does not resolve underlying drivers of OA progression in this region, such as higher mechanical loads, although no evidence of a different change in Lysholm over time was associated with having an LFC defect. Patients with trochlear defects showed the greatest decline in Lysholm scores over time, and this finding was significant when compared to patients with patellar defects. The long‐term clinical patient‐reported outcomes in a recent study of tibiofemoral versus patellofemoral MACI also were better in the tibiofemoral treated patients, despite similar magnetic resonance imaging‐based outcomes across groups [6].

Defect grade was significantly associated with longitudinal outcome after ACI, with grade 4 defects showing a 1.0 point/year faster decline in Lysholm score over time compared to grade 3 defects. Hence, patients with more severe defects were less likely to maintain their restored knee function than those with a lower grade severity. Our data also suggest that outcomes in patients who have received other procedures with ACI, such as a realignment surgery or a meniscal allograft, are no different from those who have isolated ACI (as the presence of a coincidental procedure was not significantly associated with the outcome at 1 year or longitudinally). This suggests that patient outcomes can be normalised if associated pathologies are corrected at the time of the index surgery, as also shown by others [18].

There are limitations that need to be considered when interpreting the results of this study. Most patients (276/290) provided postoperative Lysholm scores at 1 year. However, there was a gradual decline in response rates as time progressed and gaps in the data may have impacted the observed trends. Linear mixed effect modelling naturally handles missing longitudinal data and is one of the two methods recommended by the European Medicines Agency. However, this method only gives unbiased estimates if data are missing at random, but several scenarios are possible where missingness is related to the outcome. Older studies on patients who were lost to follow‐up after knee arthroplasty suggested that those patients had poorer outcomes [26]. However, more recent orthopaedic studies where patients who did not return questionnaires were contacted found only a very small difference in reported outcomes [7, 34, 35]. A further source of ‘missingness’ was missing baseline characteristics, which was addressed by imputing mean values, but it must be noted that findings from imputed data might not reflect that of the ‘true’ patient population. However, mean imputation provides conservative estimates.

Finally, in developing the OsCell database, some information was never collected that would have been of interest in hindsight, such as an objective measure of malalignment. Furthermore, when the database was developed the Lysholm scale was well‐established and seemed an appropriate scoring system. Nowadays, the Knee injury and Osteoarthritis Outcome Score (KOOS) has been more widely adopted, but our database predates even the first KOOS publication from 1998 [33].

## CONCLUSION

In conclusion, a complex ‘real world’ data set from a large number of ACI‐treated patients (under a range of historical treatment guidelines) was analysed, leading to the inclusion of patients that would not necessarily be treated with ACI today. Our analysis gives additional support to the well‐established positive association of lower patient age and higher preoperative scores with higher postoperative Lysholm scores but also found novel associations such as a lower number of implanted cells being associated with higher postoperative scores and the association of defect location and grade with long‐term scores. These areas should be explored more thoroughly, for instance, by experimentally varying cell numbers or making better use of imaging, to gain an enhanced understanding of how defect characteristics influence outcome.

The variables associated with ACI outcomes identified in the current study apply to a patient population representing a typical orthopaedic clinic (rather than a clinical trial setting with a more restricted cohort of patients) and will, therefore, benefit ACI surgeons in improved patient selection in both settings.

## AUTHORS CONTRIBUTIONS

All listed authors contributed to this work (Karina T. Wright, Sally Roberts and Jan H. Kuiper were involved in the study design and Jan H. Kuiper was involved in the design of the statistical analysis with some support from Mateus B. Harrington. All data analysis was conducted by Lauren Tierney. The manuscript was drafted by Lauren Tierney and all authors reviewed and edited where appropriate, (with major edits conducted by Jan H. Kuiper, Karina T. Wright and Sally Roberts) and approved the final submission to KSSTA.

## CONFLICT OF INTEREST STATEMENT

The authors declare no conflict of interest.

## ETHICS STATEMENT

Informed consent was received according to ethical approvals for the study (REACT 09/H1203/90).

## Supporting information

Supporting information.

## Data Availability

Raw data will be made available to collaborating institutions on request where appropriate.

## References

[1] Bhattacharjee, A. , McCarthy, H.S. , Tins, B. , Roberts, S. , Kuiper, J.H. , Harrison, P.E. et al. (2016) Autologous bone plug supplemented with autologous chondrocyte implantation in osteochondral defects of the knee. The American Journal of Sports Medicine, 44(5), 1249–1259. Available from: 10.1177/0363546516631739 26965681

[2] Bhosale, A.M. , Kuiper, J.H. , Johnson, W.E.B. , Harrison, P.E. & Richardson, J.B. (2009) Midterm to long‐term longitudinal outcome of autologous chondrocyte implantation in the knee joint: a multilevel analysis. The American Journal of Sports Medicine, 37(Suppl 1), 131–138. Available from: 10.1177/0363546509350555 19861698

[3] Brittberg, M. , Lindahl, A. , Nilsson, A. , Ohlsson, C. , Isaksson, O. & Peterson, L. (1994) Treatment of deep cartilage defects in the knee with autologous chondrocyte transplantation. New England Journal of Medicine, 331(14), 889–895. Available from: 10.1056/NEJM199410063311401 8078550

[4] Colombini, A. , Libonati, F. , Lopa, S. , Peretti, G.M. , Moretti, M. & de Girolamo, L. (2023) Autologous chondrocyte implantation provides good long‐term clinical results in the treatment of knee osteoarthritis: a systematic review. Knee Surgery, Sports Traumatology, Arthroscopy, 31(6), 2338–2348. Available from: 10.1007/s00167-022-07030-2 35716187

[5] Dugard, M.N. , Kuiper, J.H. , Parker, J. , Roberts, S. , Robinson, E. , Harrison, P. et al. (2017) Development of a tool to predict outcome of autologous chondrocyte implantation. Cartilage, 8(2), 119–130. Available from: 10.1177/1947603516650002 28345413 PMC5358825

[6] Ebert, J.R. , Zheng, M. , Fallon, M. , Wood, D.J. & Janes, G.C. (2024) 10‐year prospective clinical and radiological evaluation after matrix‐induced autologous chondrocyte implantation and comparison of tibiofemoral and patellofemoral graft outcomes. The American Journal of Sports Medicine, 52(4), 977–986. Available from: 10.1177/03635465241227969 38384192 PMC10943616

[7] Endler, P. , Ekman, P. , Hellström, F. , Möller, H. & Gerdhem, P. (2020) Minor effect of loss to follow‐up on outcome interpretation in the Swedish Spine Register. European Spine Journal, 29(2), 213–220. Available from: 10.1007/s00586-019-06181-0 31781864

[8] European Medicines Agency . (2010) *Guideline on missing data in confirmatory clinical trials*. Available at: https://www.ema.europa.eu/en/documents/scientific-guideline/guideline-missing-data-confirmatory-clinical-trials_en.pdf [Accessed 18 July 2023].

[9] Foldager, C.B. , Gomoll, A.H. , Lind, M. & Spector, M. (2012) Cell seeding densities in autologous chondrocyte implantation techniques for cartilage repair. Cartilage, 3(2), 108–117. Available from: 10.1177/1947603511435522 26069624 PMC4297130

[10] Ford, C. (2015) *Understanding Q‐Q plots*. Available at: https://data.library.virginia.edu/understanding-q-q-plots/ [Accessed 18 July 2023].

[11] Gillinov, S.M. , Fosam, A. , Burroughs, P.J. , Schneble, C.A. , McLaughlin, W.M. , Moran, J. et al. (2022) Incidence, timing, and risk factors for 5‐year revision surgery after autologous chondrocyte implantation in 533 patients. The American Journal of Sports Medicine, 50(11), 2893–2899. Available from: 10.1177/03635465221111115 35916771

[12] Gursoy, S. , Akkaya, M. , Simsek, M.E. , Gursoy, M. , Dogan, M. & Bozkurt, M. (2019) Factors influencing the results in matrix‐associated autologous chondrocyte implantation: a 2−5 year follow‐up study. Journal of Clinical Medicine Research, 11(2), 137–144. Available from: 10.14740/jocmr3711 30701007 PMC6340672

[13] Harrell Jr., FE . (2015) Regression modelling strategies: with applications to linear models, logistic and ordinal regression, and survival analysis, 2nd edition, vols. 2 New York: Springer, pp. 13–30.

[14] Heinze, G. , Wallisch, C. & Dunkler, D. (2018) Variable selection—a review and recommendations for the practicing statistician. Biometrical Journal, 60(3), 431–449. Available from: 10.1002/bimj.201700067 29292533 PMC5969114

[15] Howard, J.S. & Lattermann, C. (2014) Use of preoperative patient reported outcome scores to predict outcome following autologous chondrocyte implantation. Orthopaedic Journal of Sports Medicine, 2(7), 2325967114S00050. Available from: 10.1177/2325967114S00050

[16] Jaiswal, P.K. , Bentley, G. , Carrington, R.W.J. , Skinner, J.A. & Briggs, T.W.R. (2012) The adverse effect of elevated body mass index on outcome after autologous chondrocyte implantation. The Journal of Bone and Joint Surgery. British Volume, 94B(10), 1377–1381. Available from: 10.1302/0301-620X.94B10.29388 23015564

[17] Jaiswal, P.K. , Macmull, S. , Bentley, G. , Carrington, R.W.J. , Skinner, J.A. & Briggs, T.W.R. (2009) Does smoking influence outcome after autologous chondrocyte implantation?: a case‐controlled study. The Journal of Bone and Joint Surgery. British Volume, 91B(12), 1575–1578. Available from: 10.1302/0301-620X.91B12.22879 19949119

[18] Johannes, W. , Kevin‐Arno, K. , Severin, Z. , Raphael, T. , Tilman, W. , Tobias, R. et al. (2024) Neutral to slightly undercorrected mechanical leg alignment provides superior long‐term results in patients undergoing matrix‐associated autologous chondrocyte implantation. Knee Surgery, Sports Traumatology, Arthroscopy, 32(8), 2040–2051. Available from: 10.1002/ksa.12226 38738859

[19] Jones, K.J. , Sheppard, W.L. , Arshi, A. , Hinckel, B.B. & Sherman, S.L. (2019) Articular cartilage lesion characteristic reporting is highly variable in clinical outcomes studies of the knee. Cartilage, 10(3), 299–304. Available from: 10.1177/1947603518756464 29405742 PMC6585291

[20] Kocher, M.S. , Steadman, R.J. , Briggs, K.K. , Sterett, W.I. & Hawkins, R.J. (2004) Reliability, validity, and responsiveness of the Lysholm knee scale for various chondral disorders of the knee. The Journal of Bone and Joint Surgery‐American Volume, 86(6), 1139–1145. Available from: 10.2106/00004623-200406000-00004 15173285

[21] Krishnan, S.P. , Skinner, J.A. , Bartlett, W. , Carrington, R.W.J. , Flanagan, A.M. , Briggs, T.W.R. et al. (2006) Who is the ideal candidate for autologous chondrocyte implantation? The Journal of Bone and Joint Surgery. British Volume, 88B(1), 61–64. Available from: 10.1302/0301-620X.88B1.16796 16365122

[22] Lacy, K.W. , Cracchiolo, A. , Yu, S. & Goitz, H. (2016) Medial femoral condyle cartilage defect biomechanics: effect of obesity, defect size, and cartilage thickness. The American Journal of Sports Medicine, 44(2), 409–416. Available from: 10.1177/0363546515613517 26657570

[23] Luke, S.G. (2017) Evaluating significance in linear mixed‐effects models in R. Behavior Research Methods, 49(4), 1494–1502. Available from: 10.3758/s13428-016-0809-y 27620283

[24] McCarthy, H.S. , McCall, I.W. , Williams, J.M. , Mennan, C. , Dugard, M.N. , Richardson, J.B. et al. (2018) Magnetic resonance imaging parameters at 1 year correlate with clinical outcomes up to 17 years after autologous chondrocyte implantation. Orthopaedic Journal of Sports Medicine, 6(8), 2325967118788280. Available from: 10.1177/2325967118788280 30094269 PMC6081761

[25] Minas, T. , Von Keudell, A. , Bryant, T. & Gomoll, A.H. (2014) The John Insall award: a minimum 10‐year outcome study of autologous chondrocyte implantation. Clinical Orthopaedics & Related Research, 472(1), 41–51. Available from: 10.1007/s11999-013-3146-9 23979923 PMC3889462

[26] Murray, D.W. , Britton, A.R. & Bulstrode, C.J.K. (1997) Loss to follow‐up matters. The Journal of Bone and Joint Surgery. British Volume, 79B(2), 254–257. Available from: 10.1302/0301-620X.79B2.0790254 9119852

[27] Niemeyer, P. , Laute, V. , John, T. , Becher, C. , Diehl, P. , Kolombe, T. et al. (2016) The effect of cell dose on the early magnetic resonance morphological outcomes of autologous cell implantation for articular cartilage defects in the knee: a randomized clinical trial. The American Journal of Sports Medicine, 44(8), 2005–2014. Available from: 10.1177/0363546516646092 27206690

[28] Niemeyer, P. , Porichis, S. , Salzmann, G. & Südkamp, N.P. (2012) What patients expect about autologous chondrocyte implantation (ACI) for treatment of cartilage defects at the knee joint. Cartilage, 3(1), 13–19. Available from: 10.1177/1947603511415840 26069615 PMC4297184

[29] Pareek, A. , Carey, J.L. , Reardon, P.J. , Peterson, L. , Stuart, M.J. & Krych, A.J. (2016) Long‐term outcomes after autologous chondrocyte implantation: a systematic review at mean follow‐up of 11.4 years. Cartilage, 7(4), 298–308. Available from: 10.1177/1947603516630786 27688838 PMC5029566

[30] Pers, Y.M. , Rackwitz, L. , Ferreira, R. , Pullig, O. , Delfour, C. , Barry, F. et al. (2016) Adipose mesenchymal stromal cell‐based therapy for severe osteoarthritis of the knee: a phase I dose‐escalation trial. Stem Cells Translational Medicine, 5(7), 847–856. Available from: 10.5966/sctm.2015-0245 27217345 PMC4922848

[31] Pinheiro, J.C. & Douglas, M.B. (2000) Mixed‐effects models in S and S‐PLUS, vols. 5 New York: Springer, pp. 133–197.

[32] Retzky, J.S. , Fletcher, C. , Rizy, M. , Burge, A. & Strickland, S.M. (2024) Magnetic resonance observation of cartilage repair tissue (MOCART) Scores > 55 at 6 months postoperative predict ability to achieve patient acceptable symptomatic state at minimum 1 year postoperative following autologous chondrocyte implantation for Grade IV chondral defects about the patellofemoral joint. Cartilage, 16(1), 17–23. Available from: 10.1177/19476035241244491 38613220 PMC11569683

[33] Roos, H. , Laurén M , Adalberth T , Roos EM , Jonsson K , Lohmander LS (1998) Knee osteoarthritis after meniscectomy: prevalence of radiographic changes after twenty‐one years, compared with matched controls. Arthritis & Rheumatism, 41(4), 687–693. Available from: 10.1002/1529-0131(199804)41:4<687::AID-ART16>3.0.CO;2-2 9550478

[34] Ross, L.A. , O'Rourke, S.C. , Toland, G. , MacDonald, D.J. , Clement, N.D. & Scott, C.E.H. (2022) Loss to patient‐reported outcome measure follow‐up after hip arthroplasty and knee arthroplasty: patient satisfaction, associations with non‐response, and maximizing returns. Bone & Joint Open, 3(4), 275–283. Available from: 10.1302/2633-1462.34.BJO-2022-0013.R1 35357243 PMC9044084

[35] Samade, R. , Colvell, K. & Goyal, K.S. (2021) An update on loss to follow‐up after upper extremity surgery: survey of patient responses. The Hand, 16(1), 104–109. Available from: 10.1177/1558944719840743 30947548 PMC7818034

[36] Smith, H.J. , Richardson, J.B. & Tennant, A. (2009) Modification and validation of the Lysholm knee scale to assess articular cartilage damage. Osteoarthritis and Cartilage, 17(1), 53–58. Available from: 10.1016/j.joca.2008.05.002 18556222

[37] Tegner, Y. & Lysholm, J. (1985) Rating systems in the evaluation of knee ligament injuries. Clinical Orthopaedics and Related Research, 198, 43–49. Available from: 10.1097/00003086-198509000-00007 4028566

[38] Walters, S.J. , Jacques, R.M. , dos Anjos Henriques‐Cadby, I.B. , Candlish, J. , Totton, N. & Xian, M.T.S. (2019) Sample size estimation for randomised controlled trials with repeated assessment of patient‐reported outcomes: what correlation between baseline and follow‐up outcomes should we assume? Trials, 20(1), 566. Available from: 10.1186/s13063-019-3671-2 31519202 PMC6743178

[39] Zajic, A. (2019) *Introduction to AIC—akaike information criterion*. Available at: https://towardsdatascience.com/introduction-to-aic-akaike-information-criterion-9c9ba1c96ced [Accessed 12 June 2023].

