# Supplementary material for: Lower cell number, lateral defect location and milder grade are associated with improved autologous chondrocyte implantation outcome
Source: Knee Surg Sports Traumatol Arthrosc. 2024 Aug 27;33(4):1308–20. doi: 10.1002/ksa.12433 (PMC11948170; doi:10.1002/ksa.12433)
Supplement: Supplementary file 1 — Supporting information. [file KSA-33-1308-s001.docx]

**Supplementary Tables and Figures**

**Supplementary Table 1: Frequency of Lysholm scores returned per patient.**

| *Total number of follow-up questionnaires completed* | 1 | 2 | 3 | 4 | 5 | 6 | 7 | 8 | 9 | 10 | 11 | 12 | 13 | 14 | 15 | 16 |
| --- | --- | --- | --- | --- | --- | --- | --- | --- | --- | --- | --- | --- | --- | --- | --- | --- |
| *Number of patients* | 62 | 52 | 46 | 27 | 27 | 29 | 18 | 13 | 9 | 5 | 9 | 7 | 0 | 0 | 1 | 1 |

**Supplementary Table 2: Comparing random intercept (ri) and random slope and intercept (rs) models.**

| **Model** | **npar** | **AIC** | **BIC** | **Loglikelihood** | **Deviance** | **Chisq** | **Df** | **Pr(>Chisq)** |
| --- | --- | --- | --- | --- | --- | --- | --- | --- |
| *ri* | 4 | 11122 | 11142 | -5556.6 | 11113 |  |  |  |
| *rs* | 6 | 11092 | 11123 | -5539.8 | 11080 | 33.514 | 2 | 5.279e-08 |

*Key: npar = number of parameters, AIC = Akaike information criterion, BIC = Bayesian information criterion, chisq = chi square, Df = degrees of freedom, Pr = pvalue.*

**Supplementary Table 3:** Full model output

|  | **Lysholm score** | | |
| --- | --- | --- | --- |
| *Predictors* | *Estimates* | *CI* | *p* |
| (Intercept) | 142.50 | 45.31 – 239.70 | **0.004** |
| Time | -0.83 | -13.74 – 12.08 | 0.900 (n.s.) |
| Age at op | -0.29 | -0.54 – -0.04 | **0.022** |
| Gender [Male] | 2.14 | -3.15 – 7.42 | 0.428 (n.s.) |
| BMI | -0.20 | -0.74 – 0.34 | 0.467 (n.s.) |
| Smoker [Ex] | -1.66 | -16.27 – 12.95 | 0.824 (n.s.) |
| Smoker [No] | 4.96 | -3.39 – 13.31 | 0.244 (n.s.) |
| Smoker [Unknown] | 2.61 | -6.18 – 11.41 | 0.560 (n.s.) |
| Defect Grade [1 and 2] | -5.22 | -17.09 – 6.64 | 0.388 (n.s.) |
| Defect Grade [4] | 0.39 | -5.99 – 6.76 | 0.905 (n.s.) |
| Defect Grade [Unknown] | 0.40 | -5.82 – 6.62 | 0.899 (n.s.) |
| Defect location [LFC] | 5.86 | -0.27 – 12.00 | 0.061 (n.s.) |
| Defect location [LTP and MTP] | -2.68 | -14.72 – 9.35 | 0.662 (n.s.) |
| Defect location [Patella] | -3.95 | -11.58 – 3.67 | 0.309 (n.s.) |
| Defect location [Trochlea] | 2.90 | -4.58 – 10.38 | 0.446 (n.s.) |
| Defect diameter | -0.02 | -0.35 – 0.31 | 0.895 (n.s.) |
| Number of Defects Single or Multiple [Single] | -2.41 | -8.20 – 3.38 | 0.414 (n.s.) |
| Patch type [Other] | 5.97 | -14.62 – 26.56 | 0.569 (n.s.) |
| Patch type [Periosteum] | 1.44 | -4.39 – 7.26 | 0.628 (n.s.) |
| Pre Operative Score | 0.44 | 0.30 – 0.58 | **<0.001** |
| Log cells implanted | -5.66 | -11.76 – 0.44 | 0.069 (n.s.) |
| Passage number [2] | -2.25 | -9.76 – 5.27 | 0.558 (n.s.) |
| Passage number [3 and 4] | 4.51 | -5.14 – 14.16 | 0.359 (n.s.) |
| Passage number [Unknown] | 5.42 | -11.17 – 22.01 | 0.522 (n.s.) |
| Prior Microfracture [Unknown] | -0.96 | -7.18 – 5.25 | 0.761 (n.s.) |
| Prior Microfracture [Yes] | 1.50 | -4.87 – 7.87 | 0.645 (n.s.) |
| Co incidental Yes or No [No] | -0.41 | -6.01 – 5.19 | 0.886 (n.s.) |
| Co incidental Yes or No [Unknown] | 5.79 | -3.76 – 15.34 | 0.235 (n.s.) |
| Time * age at op | 0.02 | -0.01 – 0.06 | 0.235 (n.s.) |
| Time * Gender [Male] | 0.07 | -0.71 – 0.85 | 0.859 (n.s.) |
| Time * BMI | -0.03 | -0.11 – 0.04 | 0.395 (n.s.) |
| Time * Smoker [Ex] | -0.58 | -3.05 – 1.89 | 0.648 (n.s.) |
| Time * Smoker [No] | 0.16 | -1.00 – 1.33 | 0.782 (n.s.) |
| Time * Smoker [Unknown] | -0.53 | -1.79 – 0.73 | 0.410 (n.s.) |
| Time * Defect Grade [1 and 2] | 0.99 | -0.84 – 2.83 | 0.289 (n.s.) |
| Time * Defect Grade [4] | -1.18 | -2.09 – -0.27 | **0.011** |
| Time * Defect Grade [Unknown] | -0.75 | -1.62 – 0.12 | 0.090 (n.s.) |
| Time * Defect location [LFC] | -0.32 | -1.09 – 0.45 | 0.412 (n.s.) |
| Time * Defect location [LTP and MTP] | 0.43 | -1.38 – 2.24 | 0.643 (n.s.) |
| Time * Defect location [Patella] | 1.41 | 0.19 – 2.63 | **0.024** |
| Time * Defect location [Trochlea] | -0.91 | -2.08 – 0.25 | 0.123 (n.s.) |
| Time * Defect Diameter | -0.01 | -0.06 – 0.03 | 0.553 (n.s.) |
| Time * Num Defects Single or Multiple [Single] | -0.36 | -1.23 – 0.51 | 0.420 (n.s.) |
| Time * Patch type [Other] | 0.69 | -3.07 – 4.46 | 0.718 (n.s.) |
| Time * Patch type [Periosteum] | 0.64 | -0.19 – 1.46 | 0.133 (n.s.) |
| Time * Pre Op Score 100 | 0.02 | -0.00 – 0.04 | 0.059 (n.s.) |
| Time * Cells to surgery log | -0.01 | -0.79 – 0.77 | 0.980 (n.s.) |
| Time * Passage number [2] | 0.82 | -0.10 – 1.73 | 0.079 (n.s.) |
| Time * Passage number [3 and 4] | 0.83 | -0.37 – 2.04 | 0.176 (n.s.) |
| time * Passage number [Unknown] | -0.25 | -2.00 – 1.51 | 0.783 (n.s.) |
| Time * Prior Microfracture [Unknown] | 0.05 | -0.86 – 0.96 | 0.909 (n.s.) |
| Time * Prior Microfracture [Yes] | -0.13 | -1.11 – 0.85 | 0.799 (n.s.) |
| Time * Co incidental Yes or No [No] | 0.38 | -0.46 – 1.23 | 0.376 (n.s.) |
| Time * Co incidental Yes or No [Unknown] | -0.02 | -1.24 – 1.20 | 0.977 (n.s.) |
| **Random Effects** | | | |
| σ^2^ | 172.60 | | |
| τ_00_ _OsCellNo_ | 259.60 | | |
| τ_11_ _OsCellNo.time_ | 1.74 | | |
| ρ_01_ _OsCellNo_ | -0.35 | | |
| ICC | 0.60 | | |
| N _OsCellNo_ | 306 | | |
| Observations | 1295 | | |
| Marginal R^2^ / Conditional R^2^ | 0.25 / 0.70 | | |

*Note: Passage number = passage 1 vs all other categories.*

**Supplementary Table 4:** Model output for defect location and defect location and its interaction with time (all defect locations compared to MFC).

| **Lyshom score** | | | |
| --- | --- | --- | --- |
| *Predictors* | *Estimates* | *CI* | *p* |
| (Intercept) | 64.80 | 61.26 – 68.34 | **<0.001** |
| Time | -0.18 | -0.61 – 0.25 | 0.406 (n.s.) |
| **Defect location [LFC]** | **8.82** | **2.34 – 15.31** | **0.008** |
| Defect location [LTP and MTP] | -4.53 | -16.50 – 7.44 | 0.458 (n.s.) |
| Defect location [Patella] | -6.72 | -14.06 – 0.62 | 0.073 (n.s.) |
| Defect location [Trochlea] | -1.92 | -9.14 – 5.30 | 0.602 (n.s.) |
| Time * Defect location [LFC] | -0.54 | -1.26 – 0.18 | 0.140 (n.s.) |
| Time * Defect location [LTP and MTP] | 0.65 | -1.03 – 2.32 | 0.451 (n.s.) |
| Time * Defect location [Patella] | 0.89 | -0.17 – 1.95 | 0.100 (n.s.) |
| **Time * Defect location [Trochlea]** | **-1.18** | **-2.20 – -0.17** | **0.023** |
| **Random Effects** | | |  |
| σ^2^ | 174.20 | | |
| τ_00_ _OsCellNo_ | 329.44 | | |
| τ_11_ _OsCellNo.time_ | 1.64 | | |
| ρ_01_ _OsCellNo_ | -0.18 | | |
| ICC | 0.67 | | |
| N _OsCellNo_ | 306 | | |
| Observations | 1295 | | |
| Marginal R^2^ / Conditional R^2^ | 0.04 / 0.69 | | |

**Supplementary Table 5:** Model output for defect location and defect location and its interaction with time (all defect locations compared to Patella).

| **Lysholm score** | | | |
| --- | --- | --- | --- |
| *Predictors* | *Estimates* | *CI* | *p* |
| **(Intercept)** | **58.08** | **51.65 – 64.50** | **<0.001** |
| Time | 0.71 | -0.26 – 1.68 | 0.153 (n.s.) |
| Defect location [MFC] | 6.72 | -0.62 – 14.06 | 0.073 (n.s.) |
| **Defect location [LFC]** | **15.55** | **7.13 – 23.96** | **<0.001** |
| Defect location [LTP and MTP] | 2.19 | -10.92 – 15.31 | 0.743 (n.s.) |
| Defect location [Trochlea] | 4.80 | -4.19 – 13.80 | 0.295 (n.s.) |
| Time * Defect location [MFC] | -0.89 | -1.95 – 0.17 | 0.100 (n.s.) |
| **Time * Defect location [LFC]** | **-1.43** | **-2.56 – -0.30** | **0.013** |
| Time * Defect location [LTP and MTP] | -0.24 | -2.13 – 1.65 | 0.800 (n.s.) |
| **Time * Defect location [Trochlea]** | **-2.07** | **-3.41 – -0.73** | **0.002** |
